# Ammonia–triphenyl­borane

**DOI:** 10.1107/S1600536811044503

**Published:** 2011-10-29

**Authors:** Marek Dąbrowski, Krzysztof Durka, Sergiusz Luliński, Janusz Serwatowski, Jolanta Warkocka

**Affiliations:** a Physical Chemistry Department, Faculty of Chemistry, Warsaw University of Technology, Noakowskiego 3, 00-664 Warsaw, Poland

## Abstract

The asymmetric unit of the title compound, C_18_H_18_BN or (C_6_H_5_)_3_B·NH_3_, comprises two crystallographically independent but virtually identical mol­ecules. Mol­ecules of one type are linked with each other by N—H⋯π inter­actions, generating an infinite column aligned along the *b*-axis direction. The columns of different types of mol­ecules are inter­connected by C—H⋯π inter­actions, producing a three-dimensional array.

## Related literature

For structural characterization of related triaryl­borane-ammonia complexes, see: Fuller *et al.* (2008 ▶); Hughes *et al.* (2002 ▶); Mountford *et al.* (2005 ▶).
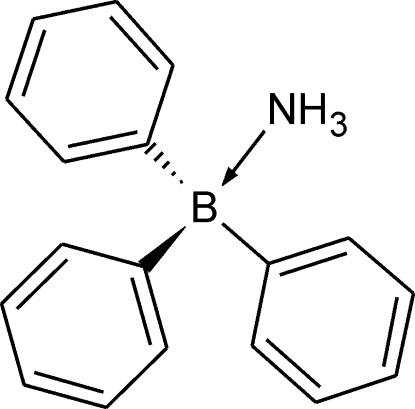


## Experimental

### 

#### Crystal data

C_18_H_18_BN
*M* *_r_* = 259.14
Monoclinic, 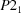

*a* = 10.3679 (5) Å
*b* = 8.8238 (4) Å
*c* = 15.7591 (8) Å
β = 90.893 (3)°
*V* = 1441.54 (12) Å^3^
*Z* = 4
Mo *K*α radiation
μ = 0.07 mm^−1^
*T* = 100 K
0.11 × 0.07 × 0.05 mm

#### Data collection

Bruker APEXII diffractometer
Absorption correction: multi-scan (*SORTAV*; Blessing, 1995 ▶) *T*_min_ = 0.972, *T*_max_ = 0.998
20884 measured reflections
7145 independent reflections
5751 reflections with *I* > 2σ(*I*)
*R*_int_ = 0.039

#### Refinement

*R*[*F* ^2^ > 2σ(*F* ^2^)] = 0.043
*wR*(*F* ^2^) = 0.096
*S* = 1.06
7145 reflections
363 parameters
1 restraint
H-atom parameters constrained
Δρ_max_ = 0.25 e Å^−3^
Δρ_min_ = −0.22 e Å^−3^

### 

Data collection: *APEX2* (Bruker, 2010 ▶); cell refinement: *SAINT* (Bruker, 2010 ▶); data reduction: *SAINT*; program(s) used to solve structure: *SHELXS97* (Sheldrick, 2008 ▶); program(s) used to refine structure: *SHELXL97* (Sheldrick, 2008 ▶); molecular graphics: *DIAMOND* (Brandenburg, 2005 ▶); software used to prepare material for publication: *PLATON* (Spek, 2009 ▶).

## Supplementary Material

Crystal structure: contains datablock(s) global, I. DOI: 10.1107/S1600536811044503/tk5004sup1.cif

Structure factors: contains datablock(s) I. DOI: 10.1107/S1600536811044503/tk5004Isup2.hkl

Additional supplementary materials:  crystallographic information; 3D view; checkCIF report

## Figures and Tables

**Table 1:** Hydrogen-bond geometry (Å, °) *Cg*1, *Cg*2, *Cg*3, *Cg*4 and *Cg*5 are the centroids of the C1–C6, C7–C12, C13–C18, C19–C24, C25–C30 and C31–C36 rings, respectively.

*D*—H⋯*A*	*D*—H	H⋯*A*	*D*⋯*A*	*D*—H⋯*A*
N1—H41⋯*Cg*1^i^	0.91	2.57	3.314 (3)	140
N1—H42⋯*Cg*2^i^	0.91	3.35	4.225 (4)	162
N1—H43⋯*Cg*3^ii^	0.91	2.70	3.553 (3)	157
N2—H44⋯*Cg*4^iii^	0.91	2.57	3.314 (3)	161
N2—H45⋯*Cg*5^iv^	0.91	2.73	3.597 (4)	160
N2—H46⋯*Cg*6^iv^	0.91	2.72	3.622 (3)	171
C4—H4⋯*Cg*5^v^	0.95	2.60	3.510 (3)	160
C8—H8⋯*Cg*4	0.95	3.01	3.844 (3)	152
C22—H22⋯*Cg*3	0.95	2.74	3.544 (3)	143

Symmetry codes: (i) 
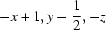
; (ii) 
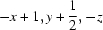
; (iii) 
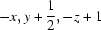
; (iv) 
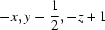
; (v) 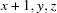.

**Fig. 1.**
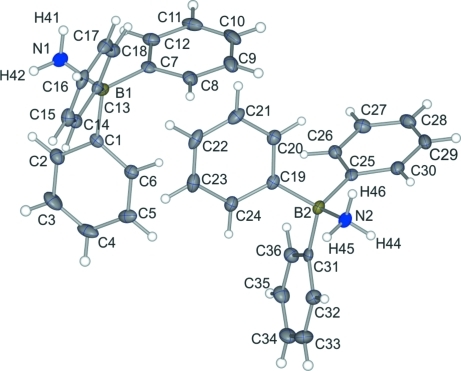
View of the title compound (I) with the atom numbering scheme. Displacement ellipsoids for non-H atoms are drawn at the 40% probability level.

**Fig. 2.**
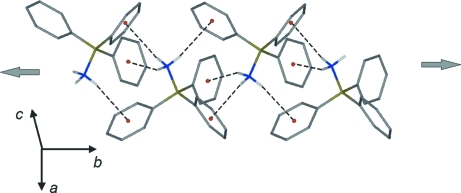
Part of the crystal structure of (I) showing formation of a column along the [010] direction. The N—H···π interactions are depicted as dashed lines. H atoms bonded to C atoms have been omitted for clarity.

**Fig. 3.**
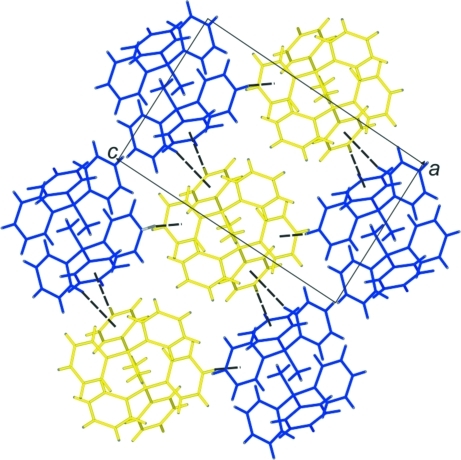
Packing diagram along b axis, indicating the columns of N—H···π interacting molecules of (I). Each column is composed of only one type of molecule coloured as yellow or blue. The C—H···π interactions are depicted as dashed lines.

## supplementary crystallographic information

### Comment

The geometries of two independent molecules, 1 and 2, differ only marginally (Fig. 1, Table 1). The B—N bond distances are slightly longer whereas the B—C bonds are slightly shorter than those found in related complexes (Fuller *et al.*, 2008; Hughes *et al.*, 2002; Mountford *et al.*, 2005). Molecules of the same type are linked by N—H··· π contacts. Two ammonia H atoms are linked to two phenyl rings of an adjacent molecule whereas the third ammonia H atom is connected to one of the phenyl rings of another molecule (Fig. 2). As a result, infinite columns are formed by molecules of one type and, separately, by molecules of the other type. N—H··· π interactions involve all ammonia H atoms and all phenyl rings. However, for columns formed by molecules 1 one of the N—H··· π contacts is significantly longer (Table 2). Columns of both types are aligned parallel to the *b* axis. The columns of 1 and 2 are linked with each other by C—H··· π interactions. For molecules of type 1, one of the *para* H atoms and one of the *ortho* H atoms are engaged into these interactions (Table 2, Fig. 3). Conversely, for molecules of type 2, only one of the *para* H atoms is connected to a phenyl ring of an adjacent molecule of type 1. Thus, a three-dimensional array is formed.

### Experimental

The title compound (I) was formed incidentally during the attempted synthesis of ammonium (4-iodophenyl)triphenyl borate. Crystals of (I) were grown by slow evaporation of its solution (0.3 g) in hexane/acetone (10 ml, 1:1).

### Refinement

All hydrogen atoms were visible in difference maps but were placed in calculated positions with C—H = 0.95 Å (phenyl) and N—H = 0.91 Å (NH_3_), and included in the refinement in the riding-model approximation with *U*_iso_(phenyl-H) = 1.2*U*_eq_(C) and *U*_iso_(NH_3_-H) = 1.5*U*_eq_(N). In the absence of significant anomalous scattering, 3602 Friedel opposites were merged so that the absolute structure was not determined.

### Crystal data

**Table:**

C_18_H_18_BN	*F*(000) = 552
*M**_r_* = 259.14	*D*_x_ = 1.194 Mg m^−^^3^
Monoclinic, *P*2_1_	Melting point: 483 K
Hall symbol: P 2yb	Mo *K*α radiation, λ = 0.71073 Å
*a* = 10.3679 (5) Å	Cell parameters from 745 reflections
*b* = 8.8238 (4) Å	θ = 2.8–29.2°
*c* = 15.7591 (8) Å	µ = 0.07 mm^−^^1^
β = 90.893 (3)°	*T* = 100 K
*V* = 1441.54 (12) Å^3^	Unspecified, colourless
*Z* = 4	0.11 × 0.07 × 0.05 mm

### Data collection

**Table:**

Bruker APEXII diffractometer	7145 independent reflections
Radiation source: TXS rotating anode	5751 reflections with *I* > 2σ(*I*)
multi-layer optics	*R*_int_ = 0.039
ω scans	θ_max_ = 27.5°, θ_min_ = 1.3°
Absorption correction: multi-scan (*SORTAV*; Blessing, 1995)	*h* = −13→13
*T*_min_ = 0.972, *T*_max_ = 0.998	*k* = −11→11
20884 measured reflections	*l* = −20→20

### Refinement

**Table:**

Refinement on *F*^2^	Primary atom site location: structure-invariant direct methods
Least-squares matrix: full	Secondary atom site location: difference Fourier map
*R*[*F*^2^ > 2σ(*F*^2^)] = 0.043	Hydrogen site location: inferred from neighbouring sites
*wR*(*F*^2^) = 0.096	H-atom parameters constrained
*S* = 1.06	*w* = 1/[σ^2^(*F*_o_^2^) + (0.0462*P*)^2^ + 0.0738*P*] where *P* = (*F*_o_^2^ + 2*F*_c_^2^)/3
7145 reflections	(Δ/σ)_max_ < 0.001
363 parameters	Δρ_max_ = 0.25 e Å^−^^3^
1 restraint	Δρ_min_ = −0.22 e Å^−^^3^

### Special details

**Table:**

Geometry. Bond distances, angles *etc*. have been calculated using the rounded fractional coordinates. All su's are estimated from the variances of the (full) variance-covariance matrix. The cell e.s.d.'s are taken into account in the estimation of distances, angles and torsion angles
Refinement. Refinement of *F*^2^ against ALL reflections. The weighted *R*-factor *wR* and goodness of fit *S* are based on *F*^2^, conventional *R*-factors *R* are based on *F*, with *F* set to zero for negative *F*^2^. The threshold expression of *F*^2^ > σ(*F*^2^) is used only for calculating *R*-factors(gt) *etc*. and is not relevant to the choice of reflections for refinement. *R*-factors based on *F*^2^ are statistically about twice as large as those based on *F*, and *R*- factors based on ALL data will be even larger.

### Fractional atomic coordinates and isotropic or equivalent isotropic displacement parameters (Å^2^)

**Table:**

	*x*	*y*	*z*	*U*_iso_*/*U*_eq_	
N1	0.46851 (13)	0.30117 (16)	−0.02663 (8)	0.0244 (4)	
C1	0.51455 (14)	0.45031 (17)	0.11530 (10)	0.0192 (4)	
C2	0.64440 (15)	0.4531 (2)	0.09392 (11)	0.0279 (5)	
C3	0.73558 (17)	0.5327 (2)	0.14187 (14)	0.0395 (6)	
C4	0.70043 (18)	0.6109 (2)	0.21272 (13)	0.0366 (6)	
C5	0.57240 (18)	0.61029 (19)	0.23632 (11)	0.0297 (6)	
C6	0.48228 (16)	0.53094 (18)	0.18834 (10)	0.0230 (5)	
C7	0.28194 (14)	0.46142 (18)	0.04095 (10)	0.0212 (5)	
C8	0.19055 (15)	0.4907 (2)	0.10358 (11)	0.0259 (5)	
C9	0.09070 (17)	0.5922 (2)	0.09175 (13)	0.0330 (6)	
C10	0.07548 (17)	0.6675 (2)	0.01537 (14)	0.0371 (7)	
C11	0.16106 (19)	0.6391 (2)	−0.04855 (13)	0.0354 (6)	
C12	0.26316 (17)	0.5383 (2)	−0.03553 (11)	0.0273 (5)	
C13	0.36994 (14)	0.19909 (18)	0.11091 (10)	0.0186 (5)	
C14	0.45190 (15)	0.13105 (18)	0.17077 (10)	0.0215 (5)	
C15	0.42616 (16)	−0.01000 (19)	0.20614 (11)	0.0266 (5)	
C16	0.31533 (16)	−0.08671 (19)	0.18238 (11)	0.0271 (5)	
C17	0.23054 (16)	−0.0224 (2)	0.12554 (11)	0.0270 (5)	
C18	0.25819 (16)	0.1181 (2)	0.09041 (11)	0.0251 (5)	
B1	0.40558 (17)	0.3555 (2)	0.06312 (11)	0.0183 (5)	
N2	−0.01770 (13)	0.62750 (15)	0.51347 (8)	0.0226 (4)	
C19	0.10881 (15)	0.54535 (18)	0.38182 (10)	0.0199 (5)	
C20	0.23486 (16)	0.4896 (2)	0.38981 (10)	0.0247 (5)	
C21	0.27217 (18)	0.3544 (2)	0.35238 (11)	0.0301 (6)	
C22	0.18552 (18)	0.2701 (2)	0.30561 (11)	0.0298 (5)	
C23	0.06082 (17)	0.32280 (19)	0.29489 (11)	0.0285 (6)	
C24	0.02403 (16)	0.45857 (19)	0.33223 (10)	0.0244 (5)	
C25	−0.04242 (15)	0.79325 (17)	0.37658 (10)	0.0191 (4)	
C26	−0.01079 (15)	0.84243 (18)	0.29506 (10)	0.0217 (5)	
C27	−0.09062 (16)	0.93634 (19)	0.24695 (11)	0.0268 (5)	
C28	−0.20765 (16)	0.9829 (2)	0.27877 (11)	0.0295 (5)	
C29	−0.24410 (16)	0.9331 (2)	0.35696 (12)	0.0305 (6)	
C30	−0.16336 (15)	0.84001 (19)	0.40522 (11)	0.0241 (5)	
C31	0.17861 (14)	0.79560 (18)	0.46940 (10)	0.0194 (4)	
C32	0.21770 (16)	0.7975 (2)	0.55431 (11)	0.0273 (5)	
C33	0.32110 (18)	0.8840 (2)	0.58398 (12)	0.0369 (6)	
C34	0.38976 (17)	0.9719 (2)	0.52863 (12)	0.0355 (6)	
C35	0.35584 (16)	0.9719 (2)	0.44405 (12)	0.0328 (6)	
C36	0.25277 (16)	0.8857 (2)	0.41534 (11)	0.0264 (5)	
B2	0.05993 (17)	0.6944 (2)	0.43198 (11)	0.0192 (5)	
H2	0.67147	0.39901	0.04520	0.0334*	
H3	0.82323	0.53266	0.12518	0.0473*	
H4	0.76304	0.66499	0.24532	0.0439*	
H5	0.54649	0.66424	0.28536	0.0357*	
H6	0.39494	0.53123	0.20570	0.0276*	
H8	0.19769	0.43852	0.15619	0.0311*	
H9	0.03209	0.61043	0.13636	0.0396*	
H10	0.00699	0.73770	0.00713	0.0445*	
H11	0.15061	0.68840	−0.10181	0.0425*	
H12	0.32182	0.52144	−0.08028	0.0327*	
H14	0.52816	0.18301	0.18802	0.0258*	
H15	0.48451	−0.05327	0.24641	0.0319*	
H16	0.29795	−0.18394	0.20542	0.0326*	
H17	0.15303	−0.07355	0.11016	0.0324*	
H18	0.19853	0.16071	0.05076	0.0301*	
H41	0.40787	0.25119	−0.05825	0.0365*	
H42	0.53640	0.23835	−0.01579	0.0365*	
H43	0.49620	0.38380	−0.05571	0.0365*	
H20	0.29672	0.54601	0.42178	0.0296*	
H21	0.35850	0.31984	0.35923	0.0361*	
H22	0.21092	0.17674	0.28092	0.0357*	
H23	0.00020	0.26627	0.26199	0.0342*	
H24	−0.06187	0.49350	0.32369	0.0293*	
H26	0.06856	0.81022	0.27170	0.0260*	
H27	−0.06487	0.96859	0.19225	0.0322*	
H28	−0.26211	1.04863	0.24666	0.0354*	
H29	−0.32542	0.96241	0.37856	0.0366*	
H30	−0.19108	0.80706	0.45935	0.0289*	
H32	0.17183	0.73720	0.59363	0.0327*	
H33	0.34426	0.88230	0.64253	0.0443*	
H34	0.45993	1.03190	0.54866	0.0426*	
H35	0.40333	1.03140	0.40517	0.0393*	
H36	0.23122	0.88751	0.35654	0.0317*	
H44	−0.04527	0.70573	0.54615	0.0339*	
H45	0.03586	0.56694	0.54474	0.0339*	
H46	−0.08685	0.57263	0.49484	0.0339*	

### Atomic displacement parameters (Å^2^)

**Table:**

	*U*^11^	*U*^22^	*U*^33^	*U*^12^	*U*^13^	*U*^23^
N1	0.0297 (8)	0.0211 (7)	0.0224 (7)	−0.0042 (6)	0.0037 (6)	−0.0002 (6)
C1	0.0193 (8)	0.0134 (7)	0.0247 (8)	−0.0014 (6)	−0.0021 (6)	0.0065 (6)
C2	0.0221 (9)	0.0250 (9)	0.0365 (10)	−0.0022 (7)	0.0010 (7)	0.0007 (8)
C3	0.0206 (9)	0.0395 (11)	0.0581 (13)	−0.0088 (9)	−0.0048 (9)	0.0057 (10)
C4	0.0344 (10)	0.0239 (10)	0.0508 (12)	−0.0080 (8)	−0.0213 (9)	0.0031 (9)
C5	0.0425 (11)	0.0162 (8)	0.0301 (10)	0.0016 (8)	−0.0132 (8)	0.0002 (7)
C6	0.0240 (8)	0.0198 (8)	0.0251 (8)	0.0002 (7)	−0.0018 (7)	0.0022 (7)
C7	0.0201 (8)	0.0165 (8)	0.0270 (8)	−0.0071 (7)	−0.0039 (6)	−0.0005 (7)
C8	0.0197 (8)	0.0256 (9)	0.0323 (9)	−0.0046 (7)	−0.0004 (7)	0.0003 (8)
C9	0.0196 (9)	0.0293 (10)	0.0500 (12)	−0.0035 (8)	−0.0012 (8)	−0.0081 (9)
C10	0.0251 (10)	0.0259 (10)	0.0597 (14)	0.0018 (8)	−0.0180 (9)	−0.0060 (9)
C11	0.0411 (11)	0.0237 (10)	0.0408 (11)	−0.0038 (8)	−0.0225 (9)	0.0023 (8)
C12	0.0295 (9)	0.0242 (9)	0.0278 (9)	−0.0041 (8)	−0.0078 (7)	0.0006 (7)
C13	0.0191 (8)	0.0168 (8)	0.0199 (8)	−0.0015 (6)	0.0053 (6)	−0.0024 (6)
C14	0.0201 (8)	0.0199 (8)	0.0245 (8)	−0.0020 (7)	0.0026 (6)	−0.0011 (7)
C15	0.0294 (9)	0.0217 (9)	0.0290 (9)	0.0044 (7)	0.0068 (7)	0.0060 (7)
C16	0.0348 (10)	0.0148 (8)	0.0323 (10)	−0.0001 (7)	0.0173 (8)	−0.0010 (7)
C17	0.0277 (9)	0.0241 (9)	0.0295 (9)	−0.0097 (8)	0.0084 (7)	−0.0062 (8)
C18	0.0251 (9)	0.0254 (9)	0.0247 (8)	−0.0064 (7)	0.0007 (7)	0.0004 (7)
B1	0.0206 (9)	0.0179 (9)	0.0165 (8)	−0.0024 (7)	0.0020 (7)	−0.0003 (7)
N2	0.0275 (7)	0.0176 (7)	0.0229 (7)	0.0000 (6)	0.0061 (6)	0.0002 (6)
C19	0.0267 (9)	0.0148 (8)	0.0182 (8)	0.0006 (7)	0.0054 (6)	0.0024 (6)
C20	0.0277 (9)	0.0223 (9)	0.0241 (8)	0.0027 (7)	0.0036 (7)	−0.0005 (7)
C21	0.0335 (10)	0.0262 (10)	0.0309 (10)	0.0078 (8)	0.0111 (8)	0.0022 (8)
C22	0.0460 (11)	0.0167 (8)	0.0271 (9)	0.0031 (8)	0.0162 (8)	0.0005 (7)
C23	0.0427 (11)	0.0177 (9)	0.0254 (9)	−0.0064 (8)	0.0072 (8)	−0.0036 (7)
C24	0.0270 (9)	0.0203 (8)	0.0261 (9)	−0.0012 (7)	0.0033 (7)	−0.0001 (7)
C25	0.0196 (8)	0.0134 (7)	0.0243 (8)	−0.0037 (6)	−0.0002 (6)	−0.0042 (6)
C26	0.0201 (8)	0.0191 (8)	0.0259 (8)	−0.0008 (7)	−0.0002 (7)	−0.0004 (7)
C27	0.0288 (9)	0.0231 (9)	0.0285 (9)	−0.0052 (7)	−0.0036 (7)	0.0035 (7)
C28	0.0289 (9)	0.0222 (9)	0.0370 (10)	0.0022 (8)	−0.0108 (8)	−0.0002 (8)
C29	0.0210 (9)	0.0308 (10)	0.0396 (11)	0.0048 (7)	−0.0033 (7)	−0.0110 (8)
C30	0.0234 (9)	0.0220 (9)	0.0269 (9)	−0.0017 (7)	0.0008 (7)	−0.0054 (7)
C31	0.0193 (8)	0.0154 (7)	0.0236 (8)	0.0060 (6)	0.0017 (6)	−0.0034 (6)
C32	0.0297 (9)	0.0262 (9)	0.0259 (9)	−0.0010 (8)	0.0005 (7)	−0.0003 (7)
C33	0.0378 (11)	0.0426 (12)	0.0301 (10)	−0.0024 (9)	−0.0076 (8)	−0.0083 (9)
C34	0.0265 (9)	0.0335 (10)	0.0464 (11)	−0.0054 (8)	−0.0051 (8)	−0.0082 (9)
C35	0.0255 (9)	0.0294 (10)	0.0434 (11)	−0.0052 (8)	0.0022 (8)	0.0006 (9)
C36	0.0241 (9)	0.0258 (9)	0.0294 (10)	−0.0023 (7)	0.0004 (7)	0.0015 (7)
B2	0.0223 (9)	0.0168 (9)	0.0185 (9)	0.0002 (7)	0.0048 (7)	−0.0008 (7)

### Geometric parameters (Å, °)

**Table:**

N1—B1	1.639 (2)	C15—H15	0.9500
N1—H42	0.9100	C16—H16	0.9500
N1—H43	0.9100	C17—H17	0.9500
N1—H41	0.9100	C18—H18	0.9500
N2—B2	1.636 (2)	C19—B2	1.620 (2)
N2—H46	0.9100	C19—C20	1.400 (2)
N2—H45	0.9100	C19—C24	1.396 (2)
N2—H44	0.9100	C20—C21	1.389 (2)
C1—C6	1.398 (2)	C21—C22	1.372 (3)
C1—C2	1.393 (2)	C22—C23	1.382 (3)
C1—B1	1.620 (2)	C23—C24	1.391 (2)
C2—C3	1.391 (3)	C25—B2	1.619 (2)
C3—C4	1.367 (3)	C25—C30	1.401 (2)
C4—C5	1.384 (3)	C25—C26	1.400 (2)
C5—C6	1.383 (2)	C26—C27	1.388 (2)
C7—C12	1.394 (2)	C27—C28	1.383 (2)
C7—C8	1.403 (2)	C28—C29	1.367 (3)
C7—B1	1.620 (2)	C29—C30	1.391 (2)
C8—C9	1.379 (2)	C31—B2	1.624 (2)
C9—C10	1.382 (3)	C31—C32	1.392 (2)
C10—C11	1.376 (3)	C31—C36	1.403 (2)
C11—C12	1.395 (3)	C32—C33	1.391 (3)
C13—C14	1.396 (2)	C33—C34	1.374 (3)
C13—B1	1.618 (2)	C34—C35	1.373 (3)
C13—C18	1.395 (2)	C35—C36	1.382 (2)
C14—C15	1.391 (2)	C20—H20	0.9500
C15—C16	1.380 (2)	C21—H21	0.9500
C16—C17	1.368 (2)	C22—H22	0.9500
C17—C18	1.390 (2)	C23—H23	0.9500
C2—H2	0.9500	C24—H24	0.9500
C3—H3	0.9500	C26—H26	0.9500
C4—H4	0.9500	C27—H27	0.9500
C5—H5	0.9500	C28—H28	0.9500
C6—H6	0.9500	C29—H29	0.9500
C8—H8	0.9500	C30—H30	0.9500
C9—H9	0.9500	C32—H32	0.9500
C10—H10	0.9500	C33—H33	0.9500
C11—H11	0.9500	C34—H34	0.9500
C12—H12	0.9500	C35—H35	0.9500
C14—H14	0.9500	C36—H36	0.9500
			
N1···C2^i^	3.448 (2)	C36···H26	3.0100
N1···C1^i^	3.402 (2)	C36···H20	3.0300
N2···C31^ii^	3.383 (2)	H2···N1	2.5300
N2···C36^ii^	3.440 (2)	H2···H42	2.2000
N2···C30^ii^	3.395 (2)	H2···H43	2.4000
N1···H12	2.6000	H2···C11^i^	2.8800
N1···H2	2.5300	H2···C17^iii^	2.9700
N2···H30	2.5300	H2···C18^iii^	2.9800
N2···H32	2.5100	H3···H9^iv^	2.2800
C1···N1^iii^	3.402 (2)	H3···C9^iv^	2.8800
C2···C11^i^	3.507 (3)	H4···C26^iv^	2.9200
C2···C18^iii^	3.416 (2)	H4···C27^iv^	2.8300
C2···N1^iii^	3.448 (2)	H4···C28^iv^	2.8700
C4···C28^iv^	3.569 (3)	H4···C25^iv^	3.0800
C6···C8	3.307 (2)	H4···C29^iv^	2.9500
C6···C14	3.553 (2)	H4···C30^iv^	3.0400
C8···C6	3.307 (2)	H6···C8	2.6600
C8···C18	3.369 (2)	H6···C7	2.9000
C11···C2^iii^	3.507 (3)	H6···C21	3.0800
C14···C6	3.553 (2)	H6···H8	2.3300
C18···C8	3.369 (2)	H8···C6	3.1000
C18···C2^i^	3.416 (2)	H8···H6	2.3300
C20···C30^ii^	3.578 (2)	H8···C13	2.8600
C20···C36	3.523 (2)	H8···C18	3.0800
C24···C32^ii^	3.411 (2)	H8···C22	2.7900
C24···C26	3.455 (2)	H8···C23	2.8200
C26···C24	3.455 (2)	H9···H3^v^	2.2800
C26···C36	3.323 (2)	H10···H18^ix^	2.4000
C28···C4^v^	3.569 (3)	H11···H27^x^	2.5600
C29···C32^vi^	3.515 (3)	H12···N1	2.6000
C30···N2^vi^	3.395 (2)	H12···C14^iii^	2.9300
C30···C20^vi^	3.578 (2)	H12···H41	2.5700
C31···N2^vi^	3.383 (2)	H12···H43	2.2100
C32···C24^vi^	3.411 (2)	H14···C1	2.6300
C32···C29^ii^	3.515 (3)	H14···C2	3.0600
C36···C20	3.523 (2)	H16···H36^xi^	2.5700
C36···N2^vi^	3.440 (2)	H17···C9^xi^	3.0300
C36···C26	3.323 (2)	H17···C10^xi^	2.8400
C1···H14	2.6300	H18···H10^x^	2.4000
C1···H42^iii^	3.0300	H18···C7	2.8000
C1···H41^iii^	2.9200	H18···C8	3.0300
C2···H42	2.7900	H18···C10^x^	3.0100
C2···H41^iii^	2.7400	H20···H34^vii^	2.5600
C2···H14	3.0600	H20···C36	3.0300
C2···H43	2.8600	H20···C31	2.6300
C3···H41^iii^	2.7600	H22···C14	3.0900
C4···H41^iii^	2.9400	H22···C17	3.0200
C4···H33^vii^	3.0900	H22···C18	3.0900
C5···H41^iii^	3.0800	H22···C15	3.0300
C5···H33^vii^	2.9000	H22···C16	3.0100
C6···H8	3.1000	H23···C27^xi^	3.0700
C6···H41^iii^	3.0600	H24···C32^ii^	3.0700
C7···H6	2.9000	H24···C25	2.7800
C7···H18	2.8000	H26···H36	2.2400
C8···H6	2.6600	H26···C36	3.0100
C8···H18	3.0300	H26···C16^viii^	3.0800
C9···H17^viii^	3.0300	H26···C19	2.9400
C9···H3^v^	2.8800	H27···H11^ix^	2.5600
C10···H17^viii^	2.8400	H27···C11^ix^	2.8800
C10···H18^ix^	3.0100	H30···H44	2.2100
C11···H2^iii^	2.8800	H30···H46	2.4000
C11···H27^x^	2.8800	H30···N2	2.5300
C12···H43	2.8000	H30···C20^vi^	2.9100
C12···H42^iii^	2.8300	H32···H45	2.1900
C12···H41	2.9700	H32···C24^vi^	3.0600
C13···H8	2.8600	H32···C28^ii^	3.0300
C14···H12^i^	2.9300	H32···C23^vi^	3.1000
C14···H22	3.0900	H32···N2	2.5100
C14···H43^i^	2.8900	H32···H44	2.3800
C15···H22	3.0300	H32···C29^ii^	2.8900
C15···H43^i^	2.6800	H33···C5^xii^	2.9000
C16···H36^xi^	2.9000	H33···C4^xii^	3.0900
C16···H22	3.0100	H34···H20^xii^	2.5600
C16···H26^xi^	3.0800	H36···C25	2.9800
C16···H43^i^	2.8300	H36···C26	2.7000
C17···H22	3.0200	H36···H16^viii^	2.5700
C17···H2^i^	2.9700	H36···C16^viii^	2.9000
C18···H8	3.0800	H36···H26	2.2400
C18···H41	3.0600	H41···C12	2.9700
C18···H22	3.0900	H41···C18	3.0600
C18···H2^i^	2.9800	H41···C1^i^	2.9200
C19···H26	2.9400	H41···C2^i^	2.7400
C20···H30^ii^	2.9100	H41···H12	2.5700
C21···H6	3.0800	H41···C4^i^	2.9400
C22···H44^ii^	2.8300	H41···C5^i^	3.0800
C22···H8	2.7900	H41···C6^i^	3.0600
C23···H44^ii^	2.7200	H41···C3^i^	2.7600
C23···H8	2.8200	H42···C2	2.7900
C23···H32^ii^	3.1000	H42···C1^i^	3.0300
C24···H32^ii^	3.0600	H42···C12^i^	2.8300
C24···H46	3.0000	H42···H2	2.2000
C24···H44^ii^	2.9500	H43···C12	2.8000
C25···H24	2.7800	H43···H2	2.4000
C25···H45^vi^	2.7200	H43···H12	2.2100
C25···H36	2.9800	H43···C14^iii^	2.8900
C25···H4^v^	3.0800	H43···C15^iii^	2.6800
C26···H4^v^	2.9200	H43···C16^iii^	2.8300
C26···H36	2.7000	H43···C2	2.8600
C27···H23^viii^	3.0700	H44···C30	2.7800
C27···H4^v^	2.8300	H44···C32	2.8500
C28···H4^v^	2.8700	H44···H30	2.2100
C28···H32^vi^	3.0300	H44···H32	2.3800
C29···H45^vi^	2.8900	H44···C22^vi^	2.8300
C29···H32^vi^	2.8900	H44···C23^vi^	2.7200
C29···H4^v^	2.9500	H44···C24^vi^	2.9500
C30···H45^vi^	2.5200	H45···C32	2.7800
C30···H44	2.7800	H45···H32	2.1900
C30···H4^v^	3.0400	H45···C25^ii^	2.7200
C30···H46	2.8600	H45···C29^ii^	2.8900
C31···H46^vi^	2.6900	H45···C30^ii^	2.5200
C31···H20	2.6300	H46···C24	3.0000
C32···H46^vi^	2.8800	H46···C30	2.8600
C32···H24^vi^	3.0700	H46···H30	2.4000
C32···H44	2.8500	H46···C31^ii^	2.6900
C32···H45	2.7800	H46···C32^ii^	2.8800
C35···H46^vi^	3.0900	H46···C35^ii^	3.0900
C36···H46^vi^	2.7900	H46···C36^ii^	2.7900
			
B1—N1—H42	109.00	C24—C19—B2	121.30 (14)
B1—N1—H43	109.00	C20—C19—B2	122.69 (14)
H41—N1—H42	109.00	C20—C19—C24	115.87 (15)
H41—N1—H43	109.00	C19—C20—C21	121.97 (16)
H42—N1—H43	109.00	C20—C21—C22	120.63 (17)
B1—N1—H41	109.00	C21—C22—C23	119.12 (16)
H44—N2—H46	109.00	C22—C23—C24	120.03 (16)
H45—N2—H46	109.00	C19—C24—C23	122.35 (15)
H44—N2—H45	109.00	C26—C25—C30	115.34 (14)
B2—N2—H44	109.00	C26—C25—B2	120.07 (14)
B2—N2—H45	109.00	C30—C25—B2	124.56 (14)
B2—N2—H46	109.00	C25—C26—C27	122.69 (15)
C2—C1—C6	115.76 (14)	C26—C27—C28	119.90 (16)
C2—C1—B1	123.74 (14)	C27—C28—C29	119.20 (16)
C6—C1—B1	120.45 (13)	C28—C29—C30	120.68 (16)
C1—C2—C3	121.91 (16)	C25—C30—C29	122.12 (16)
C2—C3—C4	120.73 (17)	C36—C31—B2	120.69 (14)
C3—C4—C5	119.12 (17)	C32—C31—C36	115.00 (14)
C4—C5—C6	119.84 (16)	C32—C31—B2	124.28 (14)
C1—C6—C5	122.64 (15)	C31—C32—C33	122.75 (16)
C12—C7—B1	124.51 (14)	C32—C33—C34	120.03 (17)
C8—C7—C12	115.59 (15)	C33—C34—C35	119.26 (17)
C8—C7—B1	119.69 (14)	C34—C35—C36	120.18 (17)
C7—C8—C9	122.56 (16)	C31—C36—C35	122.76 (16)
C8—C9—C10	120.35 (17)	C21—C20—H20	119.00
C9—C10—C11	118.95 (17)	C19—C20—H20	119.00
C10—C11—C12	120.33 (18)	C22—C21—H21	120.00
C7—C12—C11	122.19 (16)	C20—C21—H21	120.00
C14—C13—C18	115.53 (15)	C21—C22—H22	120.00
C18—C13—B1	121.61 (14)	C23—C22—H22	120.00
C14—C13—B1	122.73 (13)	C22—C23—H23	120.00
C13—C14—C15	122.49 (15)	C24—C23—H23	120.00
C14—C15—C16	119.61 (15)	C19—C24—H24	119.00
C15—C16—C17	119.85 (16)	C23—C24—H24	119.00
C16—C17—C18	119.76 (16)	C25—C26—H26	119.00
C13—C18—C17	122.73 (16)	C27—C26—H26	119.00
C3—C2—H2	119.00	C26—C27—H27	120.00
C1—C2—H2	119.00	C28—C27—H27	120.00
C4—C3—H3	120.00	C29—C28—H28	120.00
C2—C3—H3	120.00	C27—C28—H28	120.00
C3—C4—H4	120.00	C30—C29—H29	120.00
C5—C4—H4	120.00	C28—C29—H29	120.00
C6—C5—H5	120.00	C25—C30—H30	119.00
C4—C5—H5	120.00	C29—C30—H30	119.00
C1—C6—H6	119.00	C31—C32—H32	119.00
C5—C6—H6	119.00	C33—C32—H32	119.00
C9—C8—H8	119.00	C32—C33—H33	120.00
C7—C8—H8	119.00	C34—C33—H33	120.00
C10—C9—H9	120.00	C33—C34—H34	120.00
C8—C9—H9	120.00	C35—C34—H34	120.00
C9—C10—H10	121.00	C34—C35—H35	120.00
C11—C10—H10	121.00	C36—C35—H35	120.00
C12—C11—H11	120.00	C31—C36—H36	119.00
C10—C11—H11	120.00	C35—C36—H36	119.00
C11—C12—H12	119.00	C1—B1—C7	110.78 (13)
C7—C12—H12	119.00	C1—B1—C13	111.52 (13)
C15—C14—H14	119.00	N1—B1—C7	107.88 (12)
C13—C14—H14	119.00	C7—B1—C13	114.01 (13)
C14—C15—H15	120.00	N1—B1—C1	107.78 (12)
C16—C15—H15	120.00	N1—B1—C13	104.43 (12)
C15—C16—H16	120.00	C19—B2—C31	112.50 (13)
C17—C16—H16	120.00	C25—B2—C31	112.72 (13)
C16—C17—H17	120.00	N2—B2—C19	104.57 (12)
C18—C17—H17	120.00	N2—B2—C25	106.94 (12)
C13—C18—H18	119.00	N2—B2—C31	107.01 (12)
C17—C18—H18	119.00	C19—B2—C25	112.44 (13)
			
C6—C1—C2—C3	−0.7 (2)	C24—C19—C20—C21	−1.5 (2)
B1—C1—C2—C3	−178.17 (16)	B2—C19—C20—C21	174.20 (15)
C2—C1—C6—C5	0.7 (2)	C20—C19—C24—C23	1.8 (2)
B1—C1—C6—C5	178.25 (15)	B2—C19—C24—C23	−174.01 (15)
C2—C1—B1—N1	−12.7 (2)	C20—C19—B2—N2	−99.66 (17)
C2—C1—B1—C7	−130.50 (16)	C20—C19—B2—C25	144.68 (15)
C2—C1—B1—C13	101.37 (18)	C20—C19—B2—C31	16.1 (2)
C6—C1—B1—N1	169.97 (14)	C24—C19—B2—N2	75.84 (17)
C6—C1—B1—C7	52.16 (19)	C24—C19—B2—C25	−39.8 (2)
C6—C1—B1—C13	−75.97 (18)	C24—C19—B2—C31	−168.39 (14)
C1—C2—C3—C4	0.5 (3)	C19—C20—C21—C22	0.1 (3)
C2—C3—C4—C5	−0.1 (3)	C20—C21—C22—C23	1.1 (3)
C3—C4—C5—C6	0.1 (3)	C21—C22—C23—C24	−0.9 (3)
C4—C5—C6—C1	−0.4 (3)	C22—C23—C24—C19	−0.6 (3)
C12—C7—C8—C9	−1.9 (2)	C30—C25—C26—C27	2.7 (2)
B1—C7—C8—C9	173.05 (16)	B2—C25—C26—C27	−175.32 (15)
C8—C7—C12—C11	0.7 (2)	C26—C25—C30—C29	−2.1 (2)
B1—C7—C12—C11	−173.97 (16)	B2—C25—C30—C29	175.76 (15)
C8—C7—B1—N1	163.23 (14)	C26—C25—B2—N2	−168.50 (13)
C8—C7—B1—C1	−79.02 (18)	C26—C25—B2—C19	−54.28 (19)
C8—C7—B1—C13	47.7 (2)	C26—C25—B2—C31	74.17 (18)
C12—C7—B1—N1	−22.3 (2)	C30—C25—B2—N2	13.7 (2)
C12—C7—B1—C1	95.47 (18)	C30—C25—B2—C19	127.94 (16)
C12—C7—B1—C13	−137.76 (16)	C30—C25—B2—C31	−103.61 (18)
C7—C8—C9—C10	1.5 (3)	C25—C26—C27—C28	−1.1 (3)
C8—C9—C10—C11	0.3 (3)	C26—C27—C28—C29	−1.2 (3)
C9—C10—C11—C12	−1.4 (3)	C27—C28—C29—C30	1.7 (3)
C10—C11—C12—C7	0.9 (3)	C28—C29—C30—C25	0.0 (3)
C18—C13—C14—C15	1.7 (2)	C36—C31—C32—C33	1.0 (2)
B1—C13—C14—C15	−174.18 (15)	B2—C31—C32—C33	179.04 (16)
C14—C13—C18—C17	−1.2 (2)	C32—C31—C36—C35	−0.9 (2)
B1—C13—C18—C17	174.67 (15)	B2—C31—C36—C35	−179.00 (15)
C14—C13—B1—N1	95.39 (17)	C32—C31—B2—N2	11.1 (2)
C14—C13—B1—C1	−20.7 (2)	C32—C31—B2—C19	−103.23 (18)
C14—C13—B1—C7	−147.12 (15)	C32—C31—B2—C25	128.36 (16)
C18—C13—B1—N1	−80.23 (17)	C36—C31—B2—N2	−171.04 (14)
C18—C13—B1—C1	163.64 (15)	C36—C31—B2—C19	74.67 (19)
C18—C13—B1—C7	37.3 (2)	C36—C31—B2—C25	−53.8 (2)
C13—C14—C15—C16	−0.5 (3)	C31—C32—C33—C34	−0.3 (3)
C14—C15—C16—C17	−1.2 (3)	C32—C33—C34—C35	−0.7 (3)
C15—C16—C17—C18	1.7 (3)	C33—C34—C35—C36	0.8 (3)
C16—C17—C18—C13	−0.4 (3)	C34—C35—C36—C31	0.0 (3)

Symmetry codes: (i) −*x*+1, *y*−1/2, −*z*; (ii) −*x*, *y*−1/2, −*z*+1; (iii) −*x*+1, *y*+1/2, −*z*; (iv) *x*+1, *y*, *z*; (v) *x*−1, *y*, *z*; (vi) −*x*, *y*+1/2, −*z*+1; (vii) −*x*+1, *y*−1/2, −*z*+1; (viii) *x*, *y*+1, *z*; (ix) −*x*, *y*+1/2, −*z*; (x) −*x*, *y*−1/2, −*z*; (xi) *x*, *y*−1, *z*; (xii) −*x*+1, *y*+1/2, −*z*+1.

### Hydrogen-bond geometry (Å, °)

**Table:**

Cg1, Cg2, Cg3, Cg4 and Cg5 are the centroids of the [please define], [please define], [please define], [please define] and [please define] rings, respectively.

**Table:**

*D*—H···*A*	*D*—H	H···*A*	*D*···*A*	*D*—H···*A*
N1—H41···Cg1^i^	0.91	2.57	3.314 (3)	140
N1—H42···Cg2^i^	0.91	3.35	4.225 (4)	162
N1—H43···Cg3^iii^	0.91	2.70	3.553 (3)	157
N2—H44···Cg4^vi^	0.91	2.57	3.314 (3)	161
N2—H45···Cg5^ii^	0.91	2.73	3.597 (4)	160
N2—H46···Cg6^ii^	0.91	2.72	3.622 (3)	171
C4—H4···Cg5^iv^	0.95	2.60	3.510 (3)	160
C8—H8···Cg4	0.95	3.01	3.844 (3)	152
C22—H22···Cg3	0.95	2.74	3.544 (3)	143

Symmetry codes: (i) −*x*+1, *y*−1/2, −*z*; (iii) −*x*+1, *y*+1/2, −*z*; (vi) −*x*, *y*+1/2, −*z*+1; (ii) −*x*, *y*−1/2, −*z*+1; (iv) *x*+1, *y*, *z*.
